# Uronium 3-carb­oxy-4-hy­droxy­benzene­sulfonate

**DOI:** 10.1107/S1600536813029474

**Published:** 2013-10-31

**Authors:** A. Silambarasan, M. Krishna Kumar, G. Chakkaravarthi, R. Mohan Kumar, P. R. Umarani

**Affiliations:** a Department of Physics, Presidency College, Chennai 600 005, India; b Department of Physics, CPCL Polytechnic College, Chennai 600 068, India; c Kunthavai Naacchiyaar Govt. Arts College (W), Thanjavur 613 007, India

## Abstract

In the title compound, CH_5_N_2_O^+^·C_7_H_5_O_6_S^−^, the dihedral angle between the benzene ring and the mean plane of the uronium cation is 76.02 (8)°. The carboxyl group in the anion is twisted by 1.47 (9)° from the benzene ring. In the crystal, the cation is linked to the anion by weak O—H⋯O and N—H⋯O hydrogen bonds and π–π inter­actions [centroid–centroid distance = 3.8859 (8) Å], forming a three-dimensional network.

## Related literature

For the biological activity of urea derivatives, see: Sliskovic *et al.* (1999 ▶); Furlong *et al.* (2000 ▶); Houghton *et al.* (1995 ▶). For related structures, see: Krishnakumar *et al.* (2012 ▶); Sudhahar *et al.* (2013 ▶); Worsham & Busing (1969 ▶); Nelyubina *et al.* (2007 ▶).
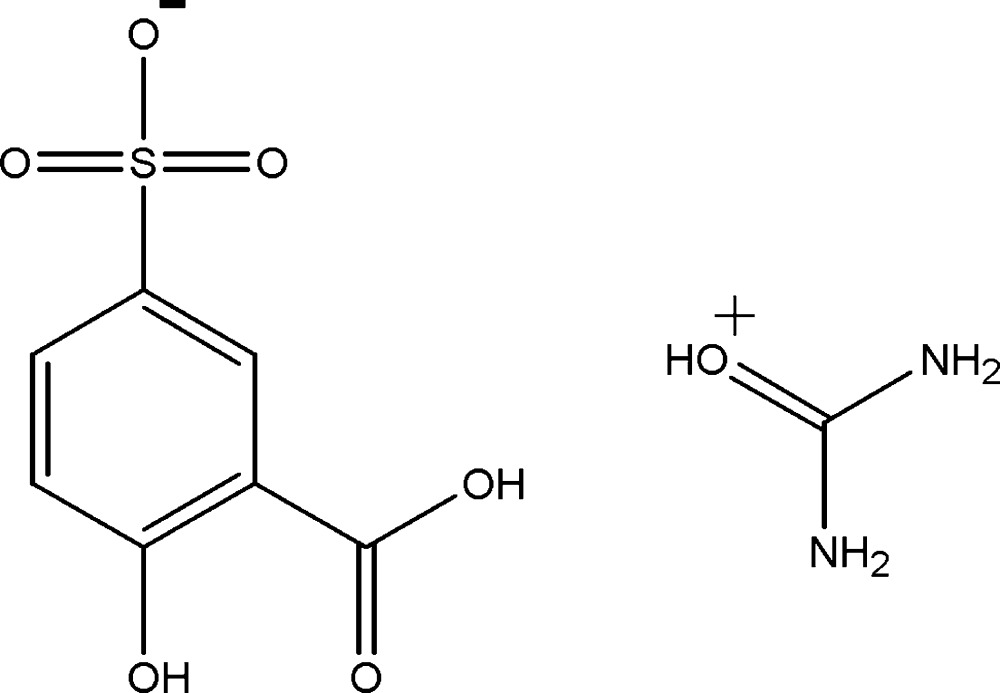


## Experimental

### 

#### Crystal data

CH_5_N_2_O^+^·C_7_H_5_O_6_S^−^
*M* *_r_* = 278.24
Triclinic, 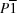

*a* = 7.2248 (4) Å
*b* = 8.7718 (5) Å
*c* = 10.1549 (5) Å
α = 85.504 (3)°
β = 71.087 (2)°
γ = 68.659 (2)°
*V* = 566.50 (5) Å^3^
*Z* = 2
Mo *K*α radiation
μ = 0.32 mm^−1^
*T* = 295 K
0.30 × 0.24 × 0.20 mm

#### Data collection

Bruker APEXII CCD diffractometer
Absorption correction: multi-scan (*SADABS*; Bruker, 2004 ▶) *T*_min_ = 0.911, *T*_max_ = 0.939
14246 measured reflections
4079 independent reflections
3432 reflections with *I* > 2σ(*I*)
*R*_int_ = 0.028

#### Refinement

*R*[*F* ^2^ > 2σ(*F* ^2^)] = 0.037
*wR*(*F* ^2^) = 0.110
*S* = 1.05
4079 reflections
185 parameters
7 restraints
H atoms treated by a mixture of independent and constrained refinement
Δρ_max_ = 0.41 e Å^−3^
Δρ_min_ = −0.40 e Å^−3^

### 

Data collection: *APEX2* (Bruker, 2004 ▶); cell refinement: *SAINT* (Bruker, 2004 ▶); data reduction: *SAINT*; program(s) used to solve structure: *SHELXS97* (Sheldrick, 2008 ▶); program(s) used to refine structure: *SHELXL97* (Sheldrick, 2008 ▶); molecular graphics: *PLATON* (Spek, 2009 ▶); software used to prepare material for publication: *SHELXL97*.

## Supplementary Material

Crystal structure: contains datablock(s) global, I. DOI: 10.1107/S1600536813029474/bh2485sup1.cif

Structure factors: contains datablock(s) I. DOI: 10.1107/S1600536813029474/bh2485Isup2.hkl

968736

Additional supplementary materials:  crystallographic information; 3D view; checkCIF report

## Figures and Tables

**Table 1:** Hydrogen-bond geometry (Å, °)

*D*—H⋯*A*	*D*—H	H⋯*A*	*D*⋯*A*	*D*—H⋯*A*
O3—H3*A*⋯O2	0.82 (1)	1.83 (1)	2.5966 (14)	154 (18)
O7—H7⋯O6	0.82 (1)	1.85 (1)	2.6629 (14)	169 (18)
N1—H1*A*⋯O4	0.84 (1)	1.96 (1)	2.7979 (16)	175 (16)
O1—H1⋯O5^i^	0.83 (1)	1.84 (1)	2.6511 (12)	167 (18)
N1—H1*B*⋯O5^ii^	0.85 (1)	1.95 (1)	2.7976 (16)	178 (15)
N2—H2*B*⋯O6^ii^	0.86 (1)	2.22 (1)	3.0712 (16)	172 (15)
N2—H2*A*⋯O3^iii^	0.85 (1)	2.19 (1)	3.0347 (16)	173 (15)

Symmetry codes: (i) 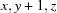; (ii) 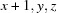; (iii) 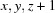.

**Fig. 1.**
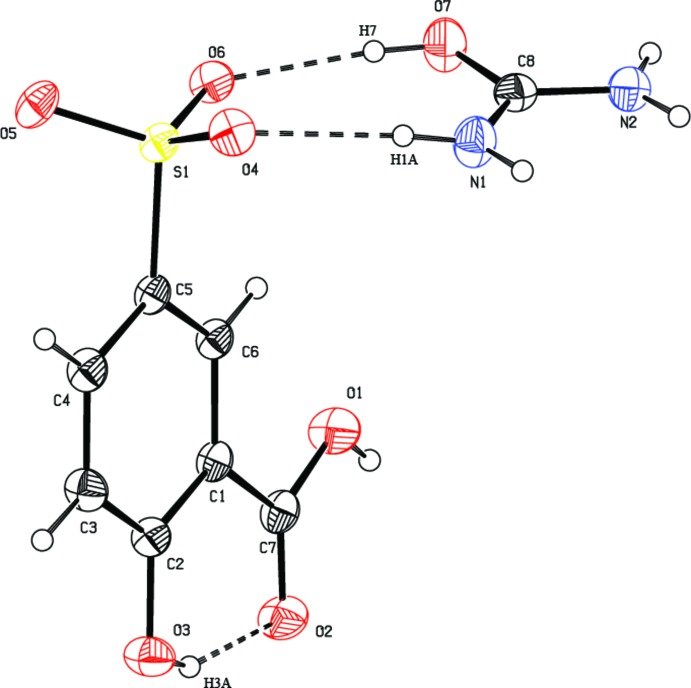
The molecular structure of (I), with 30% probability displacement ellipsoids for non-H atoms. Intramolecular hydrogen bonds are shown as dashed lines.

**Fig. 2.**
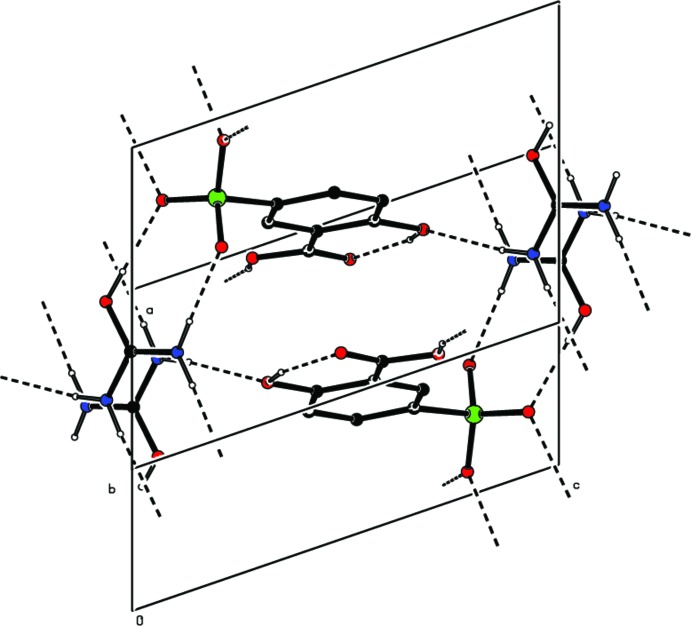
The packing of (I), viewed down b axis. Intermolecular hydrogen bonds are shown as dashed lines. H atoms not involved in hydrogen bonding have been omitted.

## supplementary crystallographic information

### 1. Comment

Urea derivatives are known to exhibit anticholesterolemic, herbicidal and antitumor activities (Sliskovic *et al.*, 1999; Furlong *et al.*, 2000; Houghton *et al.*, 1995). Herein, we report the crystal structure of the title compound (I, Fig. 1). The asymmetric unit consists of one CH_5_N_2_O^+^ cation and one C_7_H_5_O_6_S^-^ anion. In the title compound, the geometric parameters of the uronium cation (Worsham & Busing, 1969; Nelyubina *et al.*, 2007) and 3-carboxy-4-hydroxybenzenesulfonate anion (Krishnakumar *et al.*, 2012; Sudhahar *et al.*, 2013) are comparable with the reported structures.

The dihedral angle between the benzene ring and the mean plane of the uronium fragment is 76.02 (8)°. In the anion, the carboxyl group makes the dihedral angle of 1.47 (9)° with the benzene ring. In the molecular structure, cation and anion are linked by O—H···O and N—H···O hydrogen bonds, and the molecular conformation of the anion is controlled by weak O—H···O hydrogen bond (Fig. 1). The crystal structure exhibits weak intermolecular O—H···O, N—H···O (Table 1 and Fig. 2) and π···π [Cg1···Cg1^i^ distance: 3.8859 (8) Å; (*i*) *x*, 1-*y*, 1-*z*; Cg1 is the centroid of the ring C1···C6] interactions, to form a three dimensional network.

### 2. Experimental

Urea (CH_4_N_2_O, 3.003 g) and 5-sulfosalicylic acid (C_7_H_6_O_6_S, 12.711 g, 1:1 ratio) were mixed in water and the resulting solution was allowed for slow evaporation at room temperature. Good quality crystals, suitable for X-ray intensity data collection, were collected after 2 weeks.

### 3. Refinement

H atoms bonded to O and N heteroatoms were placed from difference maps and refined with free coordinates, although restrictions were applied on bond lengths: N—H = 0.86 (1) Å and O—H = 0.82 (1) Å. Isotropic displacement parameters for these H atoms were calculated as *U*_iso_(H) = 1.5*U*_eq_(O) for O—H bonds and *U*_iso_(H) = 1.2*U*_eq_(N) for N—H bonds. H atoms for CH groups were positioned geometrically and refined using a riding model, with C—H = 0.93 Å and *U*_iso_(H) = 1.2*U*_eq_(C).

### Crystal data

**Table:**

CH_5_N_2_O^+^·C_7_H_5_O_6_S^−^	*Z* = 2
*M**_r_* = 278.24	*F*(000) = 288
Triclinic, *P*1	*D*_x_ = 1.631 Mg m^−^^3^
Hall symbol: -P 1	Mo *K*α radiation, λ = 0.71073 Å
*a* = 7.2248 (4) Å	Cell parameters from 7318 reflections
*b* = 8.7718 (5) Å	θ = 2.1–32.8°
*c* = 10.1549 (5) Å	µ = 0.32 mm^−^^1^
α = 85.504 (3)°	*T* = 295 K
β = 71.087 (2)°	Block, colourless
γ = 68.659 (2)°	0.30 × 0.24 × 0.20 mm
*V* = 566.50 (5) Å^3^	

### Data collection

**Table:**

Bruker APEXII CCD diffractometer	4079 independent reflections
Radiation source: fine-focus sealed tube	3432 reflections with *I* > 2σ(*I*)
Graphite monochromator	*R*_int_ = 0.028
ω and φ scan	θ_max_ = 33.6°, θ_min_ = 2.1°
Absorption correction: multi-scan (*SADABS*; Bruker, 2004)	*h* = −11→11
*T*_min_ = 0.911, *T*_max_ = 0.939	*k* = −13→13
14246 measured reflections	*l* = −14→15

### Refinement

**Table:**

Refinement on *F*^2^	Secondary atom site location: difference Fourier map
Least-squares matrix: full	Hydrogen site location: inferred from neighbouring sites
*R*[*F*^2^ > 2σ(*F*^2^)] = 0.037	H atoms treated by a mixture of independent and constrained refinement
*wR*(*F*^2^) = 0.110	*w* = 1/[σ^2^(*F*_o_^2^) + (0.0577*P*)^2^ + 0.1089*P*] where *P* = (*F*_o_^2^ + 2*F*_c_^2^)/3
*S* = 1.05	(Δ/σ)_max_ < 0.001
4079 reflections	Δρ_max_ = 0.41 e Å^−^^3^
185 parameters	Δρ_min_ = −0.40 e Å^−^^3^
7 restraints	Extinction correction: *SHELXL97*, Fc^*^=kFc[1+0.001xFc^2^λ^3^/sin(2θ)]^-1/4^
0 constraints	Extinction coefficient: 0.105 (7)
Primary atom site location: structure-invariant direct methods	

### Fractional atomic coordinates and isotropic or equivalent isotropic displacement parameters (Å^2^)

**Table:**

	*x*	*y*	*z*	*U*_iso_*/*U*_eq_	
C1	0.22603 (17)	0.52320 (13)	0.56728 (12)	0.0301 (2)	
C2	0.28443 (18)	0.45643 (14)	0.43261 (12)	0.0324 (2)	
C3	0.3015 (2)	0.29542 (15)	0.41388 (13)	0.0376 (3)	
H3	0.3363	0.2524	0.3246	0.045*	
C4	0.26724 (19)	0.20039 (14)	0.52622 (13)	0.0347 (2)	
H4	0.2801	0.0928	0.5132	0.042*	
C5	0.21286 (17)	0.26557 (12)	0.66054 (12)	0.0288 (2)	
C6	0.19033 (17)	0.42571 (13)	0.68131 (12)	0.0301 (2)	
H6	0.1514	0.4687	0.7712	0.036*	
C7	0.20231 (19)	0.69528 (14)	0.58615 (14)	0.0343 (2)	
C8	0.5164 (2)	0.26399 (16)	1.00316 (13)	0.0378 (3)	
N1	0.6134 (2)	0.16284 (17)	0.89415 (14)	0.0474 (3)	
N2	0.6150 (2)	0.32507 (16)	1.05987 (13)	0.0431 (3)	
O1	0.1437 (2)	0.74360 (11)	0.71788 (11)	0.0490 (3)	
O2	0.23382 (18)	0.78366 (11)	0.48953 (11)	0.0473 (2)	
O3	0.32586 (18)	0.54310 (12)	0.31773 (10)	0.0459 (2)	
O4	0.39451 (15)	0.02256 (11)	0.79035 (11)	0.0417 (2)	
O5	0.05090 (15)	0.05458 (11)	0.78561 (11)	0.0417 (2)	
O6	0.08949 (15)	0.24191 (11)	0.92865 (9)	0.0380 (2)	
O7	0.31385 (16)	0.31219 (15)	1.06119 (12)	0.0513 (3)	
S1	0.18626 (4)	0.13680 (3)	0.80111 (3)	0.03090 (10)	
H1A	0.551 (2)	0.1153 (18)	0.8654 (16)	0.037*	
H1B	0.7463 (14)	0.1303 (19)	0.8589 (15)	0.037*	
H2A	0.543 (2)	0.3875 (17)	1.1326 (12)	0.037*	
H2B	0.7490 (14)	0.2929 (19)	1.0274 (16)	0.037*	
H1	0.129 (3)	0.8404 (12)	0.7270 (18)	0.046*	
H3A	0.303 (3)	0.6342 (14)	0.3493 (18)	0.046*	
H7	0.253 (3)	0.278 (2)	1.0221 (17)	0.046*	

### Atomic displacement parameters (Å^2^)

**Table:**

	*U*^11^	*U*^22^	*U*^33^	*U*^12^	*U*^13^	*U*^23^
C1	0.0310 (5)	0.0228 (4)	0.0369 (6)	−0.0100 (4)	−0.0100 (4)	−0.0027 (4)
C2	0.0333 (5)	0.0287 (5)	0.0338 (6)	−0.0102 (4)	−0.0095 (4)	−0.0006 (4)
C3	0.0466 (6)	0.0317 (5)	0.0328 (6)	−0.0125 (5)	−0.0105 (5)	−0.0068 (4)
C4	0.0412 (6)	0.0245 (5)	0.0382 (6)	−0.0114 (4)	−0.0110 (5)	−0.0070 (4)
C5	0.0300 (4)	0.0222 (4)	0.0341 (5)	−0.0099 (4)	−0.0083 (4)	−0.0037 (4)
C6	0.0342 (5)	0.0237 (4)	0.0327 (5)	−0.0115 (4)	−0.0082 (4)	−0.0053 (4)
C7	0.0361 (5)	0.0244 (5)	0.0434 (6)	−0.0117 (4)	−0.0123 (5)	−0.0015 (4)
C8	0.0418 (6)	0.0372 (6)	0.0358 (6)	−0.0163 (5)	−0.0127 (5)	0.0067 (5)
N1	0.0385 (6)	0.0541 (7)	0.0480 (7)	−0.0165 (5)	−0.0089 (5)	−0.0101 (5)
N2	0.0458 (6)	0.0439 (6)	0.0433 (6)	−0.0191 (5)	−0.0152 (5)	0.0014 (5)
O1	0.0766 (7)	0.0265 (4)	0.0465 (5)	−0.0241 (5)	−0.0149 (5)	−0.0053 (4)
O2	0.0630 (6)	0.0306 (4)	0.0512 (6)	−0.0213 (4)	−0.0176 (5)	0.0064 (4)
O3	0.0634 (6)	0.0374 (5)	0.0356 (5)	−0.0196 (5)	−0.0130 (4)	0.0036 (4)
O4	0.0428 (5)	0.0284 (4)	0.0487 (5)	−0.0056 (4)	−0.0155 (4)	−0.0018 (4)
O5	0.0445 (5)	0.0310 (4)	0.0515 (5)	−0.0216 (4)	−0.0064 (4)	−0.0068 (4)
O6	0.0445 (5)	0.0331 (4)	0.0337 (4)	−0.0155 (4)	−0.0052 (3)	−0.0066 (3)
O7	0.0402 (5)	0.0632 (7)	0.0490 (6)	−0.0176 (5)	−0.0095 (4)	−0.0137 (5)
S1	0.03538 (15)	0.02207 (13)	0.03448 (16)	−0.01185 (10)	−0.00736 (11)	−0.00307 (9)

### Geometric parameters (Å, º)

**Table:**

C1—C6	1.3966 (16)	C8—O7	1.3029 (16)
C1—C2	1.4010 (16)	C8—N1	1.3047 (17)
C1—C7	1.4766 (15)	C8—N2	1.3104 (18)
C2—O3	1.3523 (15)	N1—H1A	0.841 (9)
C2—C3	1.3951 (17)	N1—H1B	0.852 (9)
C3—C4	1.3695 (18)	N2—H2A	0.850 (9)
C3—H3	0.9300	N2—H2B	0.858 (9)
C4—C5	1.3976 (15)	O1—H1	0.826 (9)
C4—H4	0.9300	O3—H3A	0.824 (9)
C5—C6	1.3791 (14)	O4—S1	1.4468 (10)
C5—S1	1.7544 (12)	O5—S1	1.4602 (9)
C6—H6	0.9300	O6—S1	1.4654 (9)
C7—O2	1.2165 (16)	O7—H7	0.821 (9)
C7—O1	1.3189 (16)		
			
C6—C1—C2	119.27 (10)	O1—C7—C1	113.41 (11)
C6—C1—C7	121.23 (10)	O7—C8—N1	121.64 (12)
C2—C1—C7	119.50 (11)	O7—C8—N2	115.88 (12)
O3—C2—C3	117.87 (11)	N1—C8—N2	122.48 (13)
O3—C2—C1	122.19 (10)	C8—N1—H1A	121.2 (11)
C3—C2—C1	119.95 (11)	C8—N1—H1B	120.5 (11)
C4—C3—C2	120.39 (11)	H1A—N1—H1B	117.5 (15)
C4—C3—H3	119.8	C8—N2—H2A	117.9 (11)
C2—C3—H3	119.8	C8—N2—H2B	118.8 (11)
C3—C4—C5	119.82 (10)	H2A—N2—H2B	123.0 (15)
C3—C4—H4	120.1	C7—O1—H1	112.4 (12)
C5—C4—H4	120.1	C2—O3—H3A	103.8 (13)
C6—C5—C4	120.59 (11)	C8—O7—H7	115.2 (13)
C6—C5—S1	120.71 (9)	O4—S1—O5	112.06 (6)
C4—C5—S1	118.63 (8)	O4—S1—O6	112.49 (6)
C5—C6—C1	119.95 (10)	O5—S1—O6	111.64 (5)
C5—C6—H6	120.0	O4—S1—C5	106.92 (6)
C1—C6—H6	120.0	O5—S1—C5	106.35 (6)
O2—C7—O1	123.37 (11)	O6—S1—C5	106.94 (5)
O2—C7—C1	123.22 (11)		
			
C6—C1—C2—O3	−178.47 (11)	C7—C1—C6—C5	179.73 (10)
C7—C1—C2—O3	1.84 (18)	C6—C1—C7—O2	179.30 (12)
C6—C1—C2—C3	1.51 (17)	C2—C1—C7—O2	−1.02 (18)
C7—C1—C2—C3	−178.18 (11)	C6—C1—C7—O1	−0.71 (16)
O3—C2—C3—C4	178.12 (12)	C2—C1—C7—O1	178.98 (11)
C1—C2—C3—C4	−1.86 (19)	C6—C5—S1—O4	−106.66 (10)
C2—C3—C4—C5	0.64 (19)	C4—C5—S1—O4	70.32 (10)
C3—C4—C5—C6	0.94 (18)	C6—C5—S1—O5	133.45 (9)
C3—C4—C5—S1	−176.05 (10)	C4—C5—S1—O5	−49.56 (10)
C4—C5—C6—C1	−1.28 (17)	C6—C5—S1—O6	14.04 (11)
S1—C5—C6—C1	175.65 (8)	C4—C5—S1—O6	−168.98 (9)
C2—C1—C6—C5	0.05 (17)		

### Hydrogen-bond geometry (Å, º)

**Table:**

*D*—H···*A*	*D*—H	H···*A*	*D*···*A*	*D*—H···*A*
O3—H3*A*···O2	0.82 (1)	1.83 (1)	2.5966 (14)	154 (18)
O7—H7···O6	0.82 (1)	1.85 (1)	2.6629 (14)	169 (18)
N1—H1*A*···O4	0.84 (1)	1.96 (1)	2.7979 (16)	175 (16)
O1—H1···O5^i^	0.83 (1)	1.84 (1)	2.6511 (12)	167 (18)
N1—H1*B*···O5^ii^	0.85 (1)	1.95 (1)	2.7976 (16)	178 (15)
N2—H2*B*···O6^ii^	0.86 (1)	2.22 (1)	3.0712 (16)	172 (15)
N2—H2*A*···O3^iii^	0.85 (1)	2.19 (1)	3.0347 (16)	173 (15)

Symmetry codes: (i) *x*, *y*+1, *z*; (ii) *x*+1, *y*, *z*; (iii) *x*, *y*, *z*+1.

## References

[bb1] Bruker (2004). *APEX2*, *SAINT* and *SADABS* Bruker AXS Inc., Madison, Wisconsin, USA.

[bb2] Furlong, E. T., Burkhardt, M. R., Gates, P. M., Werner, S. L. & Battaglin, W. A. (2000). *Sci. Total Environ.* **248**, 135–146. 10.1016/s0048-9697(99)00537-9 10805234

[bb3] Houghton, P. J., Sosinski, J., Thakar, J. H., Border, G. B. & Grindey, G. B. (1995). *Biochem. Pharmacol.* **49**, 661–668. 10.1016/0006-2952(94)00501-c 7887981

[bb4] Krishnakumar, M., Sudhahar, S., Silambarasan, A., Chakkaravarthi, G. & Mohankumar, R. (2012). *Acta Cryst.* E**68**, o3268. 10.1107/S1600536812044509 PMC3588818 23468783

[bb5] Nelyubina, Y. V., Lyssenko, K. A., Golovanov, D. G. & Antipin, Yu. A. (2007). *CrystEngComm* **9**, 991–996.

[bb6] Sheldrick, G. M. (2008). *Acta Cryst.* A**64**, 112–122. 10.1107/S0108767307043930 18156677

[bb7] Sliskovic, D. R., Krause, B. R. & Bocan, T. M. A. (1999). *Annu. Rep. Med. Chem.* **34**, 101–110.

[bb8] Spek, A. L. (2009). *Acta Cryst.* D**65**, 148–155. 10.1107/S090744490804362X PMC2631630 19171970

[bb9] Sudhahar, S., Krishnakumar, M., Sornamurthy, B. M., Chakkaravarthi, G. & Mohankumar, R. (2013). *Acta Cryst.* E**69**, o279. 10.1107/S1600536813001785 PMC3569806 23424552

[bb10] Worsham, J. E. & Busing, W. R. (1969). *Acta Cryst.* B**25**, 572–578.

